# Supplementary material for: Effects of Chewing Gum on Satiety, Appetite Regulation, Energy Intake, and Weight Loss: A Systematic Review
Source: Nutrients. 2025 Jan 25;17(3):435. doi: 10.3390/nu17030435 (PMC11821061; doi:10.3390/nu17030435)
Supplement: Supplementary file 1 [file nutrients-17-00435-s001.zip › Table S1. Search strategy.pdf]

Table S1. Search strategy

We searched in PubMed, SCOPUS, and Cochrane Central Register of Controlled Trials. The following search strategy was applied:

#1 (“chewing gum” OR “chewing-gum)

#2 (“chewing” OR “mastication”)

#3. (“appetite regulation” OR “appetite” OR “satiety regulation” OR “satiety” OR “hunger”)

#4. (“body weight” OR “body mass index” OR “weight loss” OR “waist circumference” OR “obesity”)

#5. (“energy intake” OR “caloric intake”)

PubMed. 2000 to 2024

Search	Strategy of search
1	1 and 3
2	1 and 3 and 4
3	1 and 3 and 4 and 5
4	2 and 3
5	2 and 3 and 4
6	1 and 3 and 4 and 5

Scopus. 2000 to 2024

Search	Strategy of search
1	1 and 3
2	1 and 3 and 4
3	1 and 3 and 4 and 5
4	2 and 3
5	2 and 3 and 4
6	1 and 3 and 4 and 5

Cochrane Central Register of Controlled Trials. 2000 to 2024

Search	Strategy of search
1	1 and 3
2	1 and 3 and 4
3	1 and 3 and 4 and 5
4	2 and 3
5	2 and 3 and 4
6	1 and 3 and 4 and 5
